# The prevalence of HIV-1 DNA in AIDS-related lymphoma and Kaposi Sarcoma throughout the AIDS epidemic

**DOI:** 10.1186/1750-9378-7-S1-P43

**Published:** 2012-04-19

**Authors:** Leanne C Huysentruyt, Susanna Lamers, Michael S McGrath

**Affiliations:** 1 Department of Medicine, Hematology and Oncology, University of California, San Francisco, San Francisco, CA, USA; 2 BioInfoExperts, Thibodaux, LA, USA; 3 West Coast AIDS and Cancer Specimen Resource, University of California, San Francisco, San Francisco, CA, USA

## Background

Chronic inflammation is linked to tumorigenesis for many cancer types and likely contributes to tumor development in the HIV-infected patient population. AIDS-related lymphoma (ARL) and Kaposi Sarcoma (KS), two AIDS-defining cancers, are associated with the tumor viruses EBV and KSHV, respectively. However, EBV is only detectable in ~40% of ARLs and KSHV alone is not sufficient for KS development. Recent studies have shown that HIV is localized to tumor associated macrophages (TAM), not malignant B cells, in a portion of EBV-negative ARLs suggesting, that HIV infected TAM may play a role in tumorigenesis. The goal of this research was to determine the prevalence of HIV+ ARL and KS throughout the AIDS epidemic and examine tumor associated HIV for unique genetic signatures.

## Material and methods

Whole genomic amplified DNA from ARL and KS biopsies was used for quantitative HIV gag gene amplification. The 3’ *env-LTR* segment of HIV-1 genomes from tumor and non-tumor tissues from two patients that died of ARL were sequenced and Bayesian phylogenies were inferred using BEAST. All specimens were provided by the AIDS and Cancer Specimen Resource.

## Results

Of the 119 ARL and 91 KS biopsies, 45% and 40% contained detectable HIV-1 DNA, respectively. There was a significant decrease in the prevalence of HIV-1 DNA positive ARL and KS cases in the post-HAART era (after 1996; ARL=39%, KS=16.7) as compared to pre-HAART (ARL=54%, KS=45%). Our data suggest the overall amount of HIV DNA is less in tumor biopsies from the post-HAART era. A subset of ARL contained extremely high levels of HIV-1 DNA (~1 copy/cell). In addition, visceral KS had a higher prevalence of HIV-1 DNA (51.9%) as compared to skin KS (30.7%). HIV sequence evolution analysis of metastatic ARLs revealed that HIV was compartmentalized within sites of lymphoma and was distinct from HIV present in non-lymphoma sites.

## Conclusions

The prevalence of HIV-1 DNA positive ARLs declined in the post-HAART era, but not to the same extent as KS, consistent with the incidence of both tumor types in the post-HAART era. Higher prevalence of HIV-1 DNA in visceral sites of KS and lymphoma specific-HIV sequences in sites of metastatic lymphoma suggests that HIV, especially HIV infected macrophages, may play a role in the pathogenesis of KS and ARL disease progression. Additionally, HIV-infected macrophages are a source of chronic inflammation that may further enhance tumorigenesis. Our data suggest a tumor specific form of HIV may be evolving within individuals who develop ARL.

